# Pseudo-class part prototype networks for interpretable breast cancer classification

**DOI:** 10.1038/s41598-024-60743-x

**Published:** 2024-05-06

**Authors:** Mohammad Amin Choukali, Mehdi Chehel Amirani, Morteza Valizadeh, Ata Abbasi, Majid Komeili

**Affiliations:** 1 Department of Electrical and Computer Engineering, Urmia University, Urmia, Iran; 2 Cellular and Molecular Research Center, Cellular and Molecular Medicine Research Institute, Urmia University of Medical Sciences, Urmia, Iran; 3 Department of Pathology, Faculty of Medicine, Urmia University of medical sciences, Urmia, Iran; 4 School of Computer Science, Carleton University, Ottawa, Canada

**Keywords:** Breast cancer, Biomedical engineering

## Abstract

Interpretability in machine learning has become increasingly important as machine learning is being used in more and more applications, including those with high-stakes consequences such as healthcare where Interpretability has been regarded as a key to the successful adoption of machine learning models. However, using confounding/irrelevant information in making predictions by deep learning models, even the interpretable ones, poses critical challenges to their clinical acceptance. That has recently drawn researchers’ attention to issues beyond the mere interpretation of deep learning models. In this paper, we first investigate application of an inherently interpretable prototype-based architecture, known as ProtoPNet, for breast cancer classification in digital pathology and highlight its shortcomings in this application. Then, we propose a new method that uses more medically relevant information and makes more accurate and interpretable predictions. Our method leverages the clustering concept and implicitly increases the number of classes in the training dataset. The proposed method learns more relevant prototypes without any pixel-level annotated data. To have a more holistic assessment, in addition to classification accuracy, we define a new metric for assessing the degree of interpretability based on the comments of a group of skilled pathologists. Experimental results on the BreakHis dataset show that the proposed method effectively improves the classification accuracy and interpretability by respectively $8 \%$ and $18 \%$. Therefore, the proposed method can be seen as a step toward implementing interpretable deep learning models for the detection of breast cancer using histopathology images.

## Introduction

The use of artificial intelligence (AI) in computer-aided diagnosis (CAD) systems has the potential to improve the accuracy and efficiency of cancer diagnosis, leading to better patient outcomes. AI has shown great potential in analyzing and interpreting histopathology images, including breast cancer analysis^1^ which has been identified as one of the most prevalent and dangerous cancers among women worldwide. AI models aim to accurately classify different types of benign (B) and malignant (M) cancers, assisting pathologists in making timely and accurate diagnoses^2^. CAD systems can help overcome some of the disagreements among pathologists in the classification of different types of cancer^3^. However, to have an effective influence on clinical pathology, existing models should go beyond their “black box” behavior of solely predicting image labels and provide explainable results to help pathologists better understand and interpret the model’s predictions.

Interpretability is a crucial factor in the adoption and acceptance of AI-based systems, particularly in domains such as medical diagnosis where the consequences of errors can be severe. Physicians and other end-users need to be able to understand how an AI-based CAD system arrives at its decisions or recommendations to trust and effectively utilize it in their work^4^. The absence of interpretability in such models can lead to a lack of trust and discourage their adoption, even if they exhibit high levels of accuracy^5^. The research community is actively working to address this challenge by developing and implementing interpretability techniques that make ML models more transparent.

The majority of prior studies on interpretability for histological image classification are based on using visualization techniques to highlight the regions of the image that the model is using for classification, such as heatmaps or saliency maps^4,6–9^. Another well-known approach is to locally mimic the model predictions using linear classifiers with Local-Interpretable Model-Agnostic Explanations (LIME)^10,11^. In the field of digital pathology, there are some studies that use the pathological features for interpretability by evaluating the dependency of specific histomorphological to the model predictions features^12^ and producing an explanatory tumor histology feature map^13^. There are also some use cases of feature-specific heatmaps for visualizing deep features extracted by a model^14,15^. Revealing the learned pattern by displaying the modifications applied to the input image to change the model’s prediction^16^, and highlighting the contributions of cell nuclei in the model decision by using the graph convolutional neural networks^17^ are another examples of the studies in interpretable histopathological image classification.

The importance of interpretability in deep learning models has led to the development of different approaches to achieve it. Studies on interpretability can be roughly divided into two main categories: Post-hoc interpretability methods are applied after a model has been trained and are used to explain its predictions^18–20^. Inherently interpretable models, on the other hand, which are designed to be transparent from the outset^21–25^. Post-hoc methods, however, do not provide a complete understanding of the network’s reasoning process. Saliency maps, for example, just highlight some image regions without expressing what the model computes with the corresponding image pixels^26,27^ and therefore they may even explain false reasoning. On the other hand, interpretable-by-design methods create and employ models that make decisions through a human-understandable process. One of the popular interpretable-by-design approaches is based on case-based reasoning which relies on the storage and retrieval of past cases (i.e. input samples)^28^.

Among case-based reasoning approaches, Prototypical Part Network (ProtoPNet)^29^ is particularly interesting because it is able to provide part-based reasoning, even though it only requires image-level labels for training. The method has been shown to achieve competitive performance on several benchmark datasets while providing interpretability, indicating its potential usefulness in real-world applications. In the clinical domain, ProtoPNet has been applied to digital mammography, where it is adopted to classify mass lesions of mammogram images^30–32^. In another application, it has been applied to the brain MRI scans for Alzheimer’s disease classification^33^. In addition, there are other studies that use variants of ProtoPNet in medical applications such as diagnosing COVID-19^34^, pneumonia using chest X-ray images^35^, and breast cancer whole-slide classification^36^.

The architecture of ProtoPNet consists of three parts: a backbone network and a prototype layer followed by a linear layer. The backbone network extracts feature maps from an input image. The prototype layer consists of a set of prototypes, where each prototype corresponds to a “part” of an image that is indicative of a specific class in the dataset. The prototype layer produces an interpretable representation of the input image based on the similarity of the input patched to the learned prototypes. The linear layer combines evidence from the prototypes to make a final classification, and the weights of the linear layer reflect the importance of the corresponding prototypes for the network’s decision. The reasoning process is quite similar to that of pathologists, where an input image is identified as malignant if the pathologist finds image parts that are similar to the malignant images/sub-images that they have seen before. The model’s decision can be traced back to the relevant part prototypes, making the reasoning process transparent and easy to interpret. This makes the model suitable for AI-based CAD tools by assisting pathologists to judge the model’s output with greater confidence.

Instead of employing the information that practitioners may utilize in practice, neural networks may learn shortcuts and look at irrelevant information to solve various tasks^37^. This is known as confounding, which happens when a model generates accurate predictions based on misleading information that is not causally related to the task. Failing to address the confounding issue, can have serious consequences, especially in applications such as healthcare or finance. As a result, creating new training schemes for safeguarding ML models against confounding has drawn a lot of attention. One popular approach is to guide the model’s attention to the relevant regions through data augmentation techniques^38^ or combat confounders by extracting features that account for the inherent relation between the confounders and predictions but are invariant to the confounder factors^39^. Other approaches rely on a human expert to interactively review the generated attention maps and correct them during the training phase^40^.

Despite its inherent interpretability and competitive performance on various classification tasks, it has been shown that ProtoPNet suffers from the confounding issue, too. Barnett et al.^30^ demonstrated that when ProtoPNet is applied to mammography images, it tends to declare a sample as malignant based on the surrounding healthy tissue (i.e. irrelevant background information) rather than the lesion. To address the issue, they proposed IAIA-BL^30^ which uses predefined masks for some training images, each containing the radiologist’s pixel-level annotations of where medically relevant information is in the corresponding image. IAIA-BL penalizes prototype activations on medically irrelevant regions of radiologist-annotated training mammograms. Our experiments on histopathological images confirmed that the same problem applies to histopathological images. However, adopting such an approach for histopathological images is challenging because, (1) the size of these images is very large, making pixel-level annotation a cumbersome and time-consuming task^2^, (2) for a histopathology image there may be numerous small and large medically relevant regions, and since the model may base its decision on any of these regions, or their combinations, pathologists need to specify “all” of these regions, which is laborious and largely impractical.

In this paper, we propose a new training mechanism based on the ProtoPNet architecture to address the confounding issue in the context of digital pathology applications. The proposed method will be referred to as Pseudo-Class Part Prototype Networks (PCPPN). The proposed method leverages the clustering concept, brings out the structural similarities among the training samples within each class, and assigns them to some sub-groups (i.e. clusters). The resulting sub-groups are then considered Pseudo-classes and used to reassign labels to the data. In this way, the number of image classes is implicitly increased. The proposed method, though conceptually simple, improves the classification accuracy and interpretability of ProtoPNet by 8% and 18%, respectively on the BreakHis dataset^41^, a widely-used breast histopathological dataset. It is composed of microscopic biopsy images of breast tumors including benign and malignant types. It is considered a challenging dataset due to its large size, high variability, staining artifacts, and varying image quality. The proposed mechanism leads ProtoPNet to learn more medically relevant prototypes, effectively alleviating the confounding issue, without any pixel-level human annotation. To the best of our knowledge, our approach is the first attempt to address the confounding issue of ProtoPNet without relying on pixel-level human annotations. This potentially makes it suitable for other real-world clinical applications where explainability is crucial but obtaining pixel-level annotated is difficult.

Since the proposed PCPPN implicitly increases the number of classes, we perform additional experiments to investigate the effect of the number of classes on the confounding issue of ProtoPNet. To this end, we used the CUB-200-2011 dataset^42^ which includes images of 200 bird species. The results showed that the confounding issue is more severe when the number of classes in the dataset is small, and it disappears when there is a large number of classes. This observation is consistent with the superior results of PCPPN on the BreakHis dataset where the number of classes is implicitly increased through a clustering step.

Contributions to the paper are as follows:

- We investigated the application of ProtoPNet on digital pathology for breast histopathological images classification and demonstrated its shortcomings in this field. We realized that it suffers from confounding issue, using irrelevant information for inference.
- To tackle the confounding issue, we proposed a new training scheme PCPPN, which enables ProtoPNet to learn more medically relevant prototypes through the training step, hence making more precise and interpretable predictions. The proposed scheme leverages the clustering concept to force the network to learn medically relevant prototypes and reduce the confounding.
- The proposed PCPPN method does not require any pixel-level annotation of medically relevant regions (fine-grained data), which are rare and challenging to obtain in practice. This makes the proposed method distinct from IAIA-BL and other prior work.
- We employed the pathologists’ expertise as a golden standard in our interpretability evaluations and defined a new metric, named ‘Relevancy’, calculated based on the pathologists’ scores for the amount of medically relevant information the learned prototypes contain.
- Due to the existence of disagreement between the physicians about the relevancy value of an image’s region, we used the comments of a team of pathologists to have more precise evaluations.
- We evaluated the performance of our proposed method from both classification and interpretability perspectives. The proposed PCPPN effectively improves the accuracy and interpretability respectively by 8% and 18%, compared to ProtoPNet.

## Materials and methods

### Data

In this study, we use the BreakHis dataset^41^, a widely-used breast histopathological dataset. It consists of microscopic biopsy images of breast tumors including benign and malignant types. All images were gathered from samples of 82 patients between January 2014 and December 2014 in the P &D Laboratory, in Brazil. It is composed of 7909 RGB images (with 8 bits per color channel) in four different magnifications: $40 \times , 100\times , 200\times$, and $400\times$ (i.e. objective lens $4\times , 10\times , 20\times ,$ and $40\times$, respectively), each with the size of $700 \times 460$ pixels. Based on the pathologists’ comments about the magnification factor that is practically more informative and helpful for diagnosis, we used the images captured in the lowest ($40\times$) magnification. From the existing 1995 images of this magnification, 625 images belong to the benign class and the remaining 1370 images belong to the malignant class.

### Proposed approach

**Figure 1:**
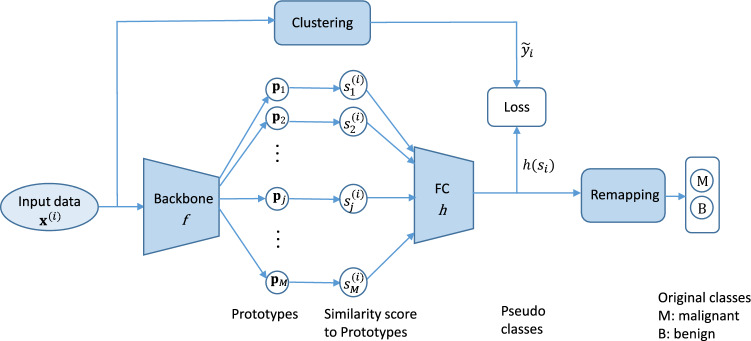
Block diagram of the proposed method.

Through our experiments, we found that as the number of classes of the dataset increases, the confounding issue of the ProtoPNet model^29^ decreases (more detail on this is provided in the Result section). However, the number of classes is often dictated by the application in hand and the corresponding dataset. Many medical diagnosis applications often are in the form of a binary classification task or involve only a few classes. Motivated by the above observation, we propose a method that aims to artificially increase the number of classes by creating a set of new classes, which we term pseudo-classes. The proposed PCPPN approach is composed of three main components: clustering, a part prototype network, and remapping. The clustering step generates pseudo-classes. The part prototype network^29^ assigns input samples to the pseudo-classes. Finally, the remapping part retrieves the actual class from the predicted pseudo-class. Figure 1 shows the block diagram of the proposed PCPPN method. In the following, each component will be described in detail.

#### Generating pseudo-classes using clustering

To increase the number of classes, we obtain a set of pseudo-classes through clustering. Clustering is done separately for the data of each class. Samples within each class are divided into *K* clusters, each represents a pseudo-class. Let *q* be the number of original classes, so there will be a total of $K \times q$ pseudo-classes, i.e. *K* pseudo-classes for each original class. Hence, for a binary classification of malignant versus benign, there will be a total of 2*K* pseudo-classes.

Data clustering algorithms, at their core, rely on a notion of distance or dissimilarity. Distance, in turn, is tied to feature space which in the simplest case could be the original space where raw data are situated. For example, the K-means clustering algorithm^43^ uses Euclidean distance. However, clustering with Euclidean distance on raw pixels is ineffective on most image datasets^44^. The common practice is to first map the original high-dimensional data to an appropriate low-dimensional space and then apply clustering. To tackle this problem for high-dimension histopathology images, the proposed method first tries to classify the training data using a black box classifier based on a deep neural network structure, and then map each data sample into its corresponding point in the learned latent space of the network. Due to its performance on the general real-world images, in this work we adopt the ResNet-18 model^45^, pre-trained on the ImageNet dataset^46^ as the black box structure. The output of the last layer before the fully connected layer of ResNet-18 is used to obtain a lower dimensional representation of the input images.

Given the pre-processd and augmented dataset, $D=\{ X,Y\}$, with images *X* and corresponding labels $Y \in \{B, M\}$, the proposed scheme first trains the ResNet-18 model to classify this dataset. The resulting network is then used as a feature extractor that maps images to their corresponding points in the resulting latent space. Then K-means clustering is applied separately to the data points of each class. After clustering, all images in the dataset are re-labeled based on the index of their corresponding cluster as new labels, i.e. pseudo-classes. The relabelled dataset is then augmented once more so that data samples have a balance distribution among the 2*K* pseudo-classes for the training process in the next stage.

#### Part prototypical network

We train a Part Prototypical structure^29^ to classify the input images to the $2\times K$ pseudo-classes obtained from the previous step. In this network, each input image *x* is first passed through a convolutional neural network (CNN) denoted by *f*, which has the same structure as the CNN mentioned earlier with the addition of two more $1 \times 1$ convolutional layers at the end. The convolution layers output a feature map, ${\textbf {z}}=f(x)$, with a shape of $7 \times 7 \times 128$ (for input image of size $224 \times 224 \times 3$). It is composed of $7 \times 7$ vectors of size $1 \times 128$, each of them represents a region of the input image in the latent space and is named a patch. In this work we used VGG-19^47^ architecture pre-trained on ImageNet as the CNN base structure.

The CNN layer *f* is followed by a prototype layer which consists of *M* prototypes $\{{\textbf {p}}_j\}_{j=1}^{M}$. Each pseudo-class is associated with a set of prototypes. Each prototype is represented by a vector of length 128 in the latent space, i.e. same size with a $1 \times 1 \times 128$ patch. Prototypes are first initialized randomly with a uniform distribution between 0 and 1, and then learned during the training process. Each prototype, after a projection step, can be seen as a representation of an image’s region in the training set. Generally, for $q \times K$ pseudo-classes, there will be $\frac{M}{q \times K}$ prototypes in each. In prototype layer, for a specific input image ${\textbf {x}}^{(i)}$ and a prototype $p_j$, an activation map of similarity scores is created based on the similarity of $p_j$ to all patches in ${\textbf {z}}^{(i)}=f({\textbf {x}}^{(i)})$. The activation map of similarity scores has a shape of $7 \times 7$, each entry represents the amount of presence of the prototype $p_j$ in the image’s region corresponding to the compared patch. A single similarity score $s_j^{(i)}$ is then obtained per prototype by applying max pooling to the activation map of similarity scores (i.e. the value of the entry with larger similarity score). $s_j^{(i)}$ takes a large value whenever there exists a region in image ${\textbf {x}}^{(i)}$ whose patch in the latent space is close to the prototype $p_j$. $s_j^{(i)}$ is inversely related to $d_j^{(i)}$, the distance between the j-th prototype and the closest patch of image ${\textbf {x}}^{(i)}$, and is calculated as $s_j^{(i)} = \log (d_j^{(i)^2}+1)/(d_j^{(i)^2}+\epsilon )$, where $\epsilon$ is a constant value equal to $1\times 10^{-4}$. In our experiments, we used Euclidean distance. The prototype layer is followed by a fully connected layer and then a softmax layer, collectively denoted as *h*, with parameters $\varvec{W}_h$.

The training process of different parts of the model is separately accomplished. First, we freeze $\varvec{W}_h$ and train the CNN and Prototype layers of the model using cross-entropy loss *CE* and two other terms as follows:

1 $$\begin{aligned} \min _{\varvec{P},\varvec{W}_c}\frac{1}{n} \sum \limits _{i=1}^{n} \left( CE(h({\textbf {s}}_i),\tilde{y}_i)+ \lambda _1 \min _{j:{\textbf {p}}_j \in {\textbf {P}}_{y_i}} d_{j}^{(i)} - \lambda _2 \min _{j:{\textbf {p}}_j \notin {\textbf {P}}_{y_i}} d_{j}^{(i)} \right) \end{aligned}$$

where $\varvec{P}$ is the set of all prototypes, $\varvec{W}_c$ denotes the CNN parameters, and ${\textbf {s}}_i$ represents the set of *M* similarity scores. The first term minimizes the cross-entropy loss between the predicted and the true pseudo-class label of i-th image, $\tilde{y}_i$. The second term encourages ${\textbf {x}}^{(i)}$ to have some latent patches that are close to a prototype associated with its true pseudo-class. The third term pushes latent patches of ${\textbf {x}}^{(i)}$ away from the prototypes associated with other pseudo-classes. $\lambda _1$ and $\lambda _2$ are hyperparameters of the model that are set as 0.8 and 0.08, respectively.

After training the CNN and prototype layers for few epochs (10 epochs), all learned prototypes are replaced with the embedding of their nearest patch from training images of the same class. Doing so, each prototype is a region of a training image which is well representative for the corresponding class. Next, the trained parameters $\varvec{W}_c$ and $\varvec{P}$ are kept frozen and the fully connected layer parameters $\varvec{W}_h$ are trained for 20 iterations. This training process continues for a predefined number of training epochs (30 epochs in this study). For more details about the Part Prototypical structure and related hyperparameters’ settings please refer to the ProtoPNet paper in^29^.

#### Remapping Pseudo-classes to the original classes

During inference, the model predicts a pseudo-class for each test sample. Since we run a separate clustering on each of the original classes, each pseudo-class is already associated with an original class. Therefore, during inference, the predicted pseudo-class is remapped to its associated original class. An overview of the proposed method is represented as pseudo-code in Algorithm 1.

**Algorithm 1:**
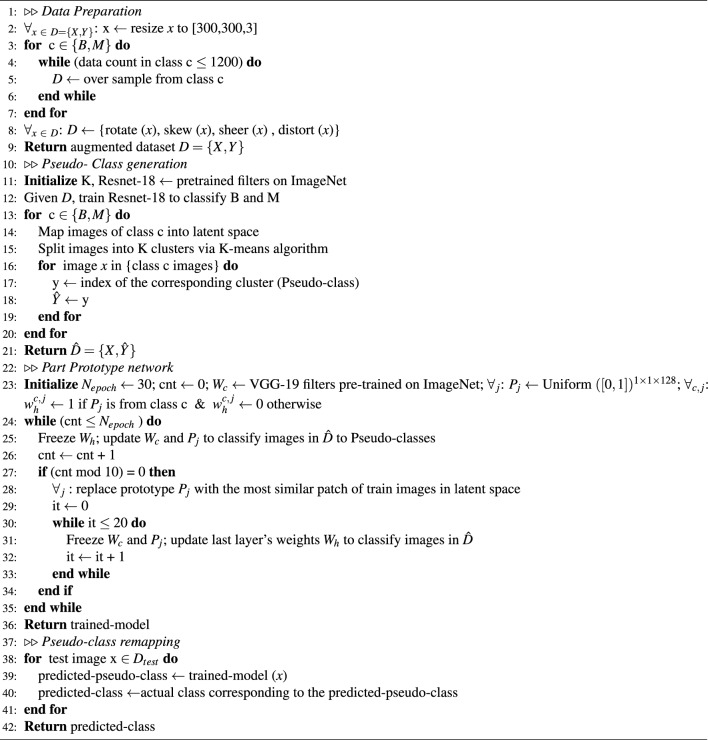
Pseudo-code of the proposed method

## Experiments and results

### Data preparation

Dealing with the $40 \times$ images of the BreakHis dataset, three pathologists were asked to verify the validity of the images’ labels of the BreakHis dataset. Pathologists reviewed the data carefully and they all identified 426 images to be mislabeled (113 images of the benign set and 313 images of the malignant set). To establish more confident experiments, we remove these images from the original dataset. We use the standard five folds provided by the dataset for train/test splits. See^41^ for more information. Removing the mislabeled images from each fold, the remaining data have class distributions in the ratio of 63–70$\%$ to 27–30$\%$ for train and test sets, respectively. All histopathology images are first normalized and resized to $300 \times 300 \times 3$. Due to the small number of training images and also the imbalanced distribution between benign and malignant classes, in each fold we first balance the number of images per class by oversampling the minority class so that there are 1200 images per class, then we augment the training data four times with random rotation, skewing, sheering, and distortion. So, there is an augmented dataset with 4800 images per class for model training.

### Evaluations

To evaluate the performance of the proposed PCPPN method, two metrics including accuracy and relevancy are used for tracking the classification ability and effectiveness in handling the confounding issue, respectively. The simulations are established through five different distributions of data, then the average values of metrics are reported. Moreover, the comments of three experienced pathologists are employed for interpretability evaluation. First, the process of making decisions by ProtoPNet structure is briefly explained to them so that they are familiar with the concept of prototypes, then they are asked to rate the relevancy level of the highlighted parts of the shown image for the determined class. To make our evaluations more reliable, the scoring processes of pathologists are independent of each other, they also do not know the relation between prototypes and experiments to avoid bias.

Inspired by the surge of adopting humans in model interpretability evaluations^48^, we try to follow this concept up clinically and use the pathologists’ comments as a gold standard for establishing a human-centric evaluation. Similar to our study, in Ref.^30^ the authors asked the radiologists to provide some fine annotated data by defining a mask per image, then they used the provided masks to direct the attention of the network to the relevant parts and evaluate the interpretability of the model by calculating the intersection between highlighted image parts by model and corresponding masks. For diagnosing breast cancer, however, providing such a mask for histopathology images is quite challenging. Besides, considering the issues of users’ prior knowledge and confirmation bias faced with the distinction and agreement criteria in HIVE^48^, we do not exploit them here because the real-world end users of the provided CAD system are the pathologists, i.e. domain experts. On the other hand, since the reasoning process of ProtoPNet is all based on looking for the learned patterns, i.e. prototypes related to each class among the input image regions, the interpretability evaluation of the model can be effectively established based on the learned prototypes.

Therefore, we propose a novel ‘Relevancy’ metric, based on the pathologists’ assessments of the prototypes from the confounding perspective. To this end, each learned prototype is visualized (as in section 2.3 of Ref.^29^) and shown to the pathologists accompanied by its corresponding activation map, then the pathologists were asked to rate the relevancy level of the highlighted parts from 0 to 3. They choose the prototype’s relevancy score from 0: Not relevant at all, (1) Slightly relevant, (2) Somewhat relevant, and (3) Completely relevant. In this study, to facilitate the rating process and make the pathologists’ evaluations more convenient and accurate, a graphical user interface (GUI) was designed to display the prototypes to physicians and gather their evaluations of the prototypes’ relevancy.

Let $v_j^{(i)}$ be the relevancy score for the jth prototype, specified by the ith pathologist, in this case, the new evaluation metric *Relevancy*, which measures the amount of medically relevant information contained in the learned prototypes, is calculated as

2 $$\begin{aligned} Relevancy = \frac{1}{M \times P}\sum \limits _{i=1}^{P} \sum \limits _{j=1}^{M} (\frac{v_j^{(i)}}{3}) \times 100 \end{aligned}$$

where, *M* and *P* are the numbers of prototypes and pathologists present in the experiments, respectively.

### Results

#### Accuracy

**Figure 2:**
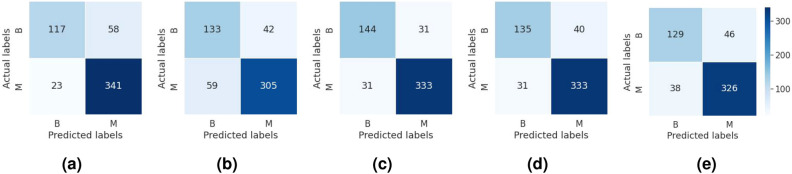
Confusion matrices produced by (**a**) ResNet-18 (black box model), (**b**) ProtoPNet, (**c**) proposed PCPPN with K = 2, (**d**) K = 4, and (**e**) K = 8 clusters (averaged over five folds). B: benign, M: malignant.

To evaluate the classification performance of our PCPPN method we train the original ProtoPNet and our model with different numbers of clusters on 5 different splits of the dataset as mentioned. The obtained classification accuracies of benign and malignant test data for all experiments are averaged and reported in Table 1. Besides, the resulting confusion matrices (averaged over five folds) of the black box model, ProtoPNet, and proposed PCPPN method are shown in Fig. 2. As can be seen, for any number of clusters considered, proposed method achieves higher average accuracy than the original ProtoPNet structure (4–8$\%$ improvement), and it achieves the best classification accuracy among others for setting K = 2 clusters with a relatively low standard deviation throughout different folds. The second rank also belongs to the PCPPN with K = 4 clusters. Besides its superiority in benign class detection accuracy, our PCPPN method is significantly more accurate in detecting the malignant class, which is very important for pathologists in practice. Moreover, comparing the performance of PCPPN with the black box base structure (ResNet-18) we can see that our interpretable method outperforms the black box model, too.

**Table 1:** Classification accuracy (in $\%$) for ProtoPNet and the proposed PCPPN method for different numbers of clusters. The best-performing interpretable method is highlighted in bold. Italic entry stands for the second-best method.

Method	Benign accuracy	Malignant accuracy	Average accuracy
ResNet-18 (black box)	68.23 ± 11.55	93.58 ± 4.30	84.87 ± 4.08
Original ProtoPNet	76.78 ± 11.98	83.41 ± 7.27	80.93 ±m 7.30
PCPPN (K = 2)	**83.24 ± 8.76**	**91.26 ± 6.36**	**88.27 ± 4.60**
PCPPN (K = 4)	*78.30 ± 8.74*	*91.07 ± 6.69*	*86.55 ± 5.47*
PCPPN (K = 8)	$74.63 \pm 11.84$	$89.67 \pm 8.30$	$84.22 \pm 4.18$

#### Relevancy

Evaluating the interpretability functionality, we conduct a human-centric study composed of experienced pathologists. For each experiment, pathologists are asked to rate the medically relevant information carried by the trained model prototypes, then the percentage of relevancy for benign and malignant classes’ prototypes is calculated separately using Eq. (2). The resulting relevancies are averaged among 5 folds and presented in Table 2. 8–18$\%$ increment of the average relevancy metric, the proposed PCPPN method has been proven to be significantly efficient in interpretability improvement. The PCPPN method with $K=8$ clusters achieves the best relevancy value on average and especially in malignant class. With $K=2$ it provides the second most medically relevant prototypes for both benign and malignant classes, while the most relevant benign prototypes are achieved by setting *K* to 4. Besides, considering the standard deviation values for relevancy reported in Table 3, it can be seen that our method with two clusters provides the second-best value of average relevancy with the lowest variation among five different experiments, and hence it could be viewed as the most reliable setting.

**Table 2:** Interpretability performance (in $\%$) for ProtoPNet and proposed PCPPN method for different numbers of clusters. The highest relevancy is highlighted in bold.

Method	Benign relevancy	Malignant relevancy	Average relevancy
Original ProtoPNet	63.61 ± 10.73	55.00 ± 29.6841	59.30 ± 20.2071
PCPPN (K = 2)	71.94 ± 9.79	75.00 ± 4.05	73.47 ± 7.24
PCPPN (K = 4)	73.33 ± 5.93	62.50 ± 18.81	67.92 ± 14.34
PCPPN (K = 8)	71.39 ± 13.49	83.89 ± 8.82	**77.64 ± 12.60**

**Table 3:** Disagreement of pathologists when rating the relevancy of prototypes provided by different methods. The lowest disagreement value in each column is highlighted in bold, while the italic text indicates the method with the second-lowest disagreement.

Method	Benign disagreement	Malignant disagreement	Average disagreement
ProtoPNet	0.8007	0.5386	0.6696
PCPPN (K =2)	**0.5699**	*0.5036*	**0.5368**
PCPPN (K =4)	*0.6555*	0.5567	0.6061
PCPPN (K =8)	0.7187	**0.4076**	*0.5632*

#### Disagreement of pathologists

As the employed interpretability metric in this work is established directly based on the pathologists’ rating, it is worth pointing out that there is an inherent disagreement between the comments of different pathologists in practice. So, diving deeper into the interpretability evaluation, besides the relevancy metric another assessment is required for more confidence. Notably, among different methods with higher medically relevant prototypes, the method that leads to the most consensus between pathologists will be more reliable and acceptable. Therefore, we choose to use the standard deviation of pathologists’ rates. For every benign and malignant prototype, the standard deviation of different pathologists’ ratings is calculated and then averaged among different experiments and reported as the disagreement value in Table 3. As it can be seen, compared to ProtoPNet, the proposed method learns medically relevant prototypes with higher consensus between pathologists, generally. Particularly, with 2 clusters it achieves the minimum disagreement (most consensus) of pathologists.

#### Repeatability

From the interpretability perspective, one method can be trustworthy whose performance does not change much when it is trained at different times with the same architecture i.e. intra-architecture repeatability^49^. To investigate the repeatability we trained the same structure three different times and evaluate the interpretability of learned prototypes each time. For different sets of learned weights, the relevancy values of the original ProtoPNet and the proposed method with two clusters are reported in Table 4. The superiority of interpretability of PCPPN has been proven due to its highest relevant learned prototypes for all sets of learned weights. It significantly improves the relevancy of learned prototypes of both benign and malignant classes on average. Despite that, the obtained relevancy values of the proposed method have much smaller standard deviations in different experiments compared to the ProtoPNet, and thus its relevancy is considerably more repeatable compared to the ProtoPNet. Moreover, the resulting average accuracies in the last column of Table 4 validate the effectiveness of our method for accuracy improvement.

**Table 4:** Repeatability analysis for interpretability of ProtoPNet and proposed PCPPN method over three different sets of learned weights (W1, W2, and W3). The lowest standard deviation values (most repeatable method) also the best relevancy and accuracy values are highlighted in bold. The last column represents the average test accuracy over different sets of learned weights.

Method	B/M	W1	W2	W3	Avg relevancy	std relevancy	Avg Acc
ProtoPNet	B	66.67	75.00	93.75	78.47	13.87	74.34
PCPPN (k=2)	B	70.83	81.25	89.58	**80.56**	**9.39**	**80.49**
ProtoPNet	M	4.16	62.50	70.83	45.83	36.32	89.34
PCPPN (k=2)	M	79.17	70.83	83.33	**77.78**	**6.36**	**89.64**
ProtoPNet	all	35.12	68.75	82.29	62.15	24.12	83.42
PCPPN (k=2)	all	75.00	76.04	86.46	**79.17**	**6.34**	**87.55**

#### Reproducibility

We investigated the performance using different backbone network architectures. This indicates if the amount of the model’s interpretability is architectures-dependent or architectures-agnostic. Table 5 represents the resulting relevancy values of ProtoPNet and PCPPN with two clusters for three different base architectures Vgg-19, ResNet-18, and DenseNet-121^50^. As we can see, the proposed method achieves a higher relevancy value of all learned prototypes for every base structure, meanwhile, the calculated standard deviations of resulting relevancy values through different base architectures for our method are much smaller than ProtoPNet, hence it has higher reproducibility compared to ProtoPNet. Moreover, from the last column of Table 5 it can be seen that the PCPPN method outperforms the original ProtoPNet in terms of the test accuracy on average, particularly in the case of malignant class. Besides, our method effectively increases the test accuracy on average, particularly for malignant class.

**Table 5:** Reproducibility analysis for interpretability of ProtoPNet and the proposed PCPPN method over three different base architectures. The lowest standard deviation over different base architectures (most reproducible method) and the best average relevancy are shown in bold. Also, the average test accuracy over different base architectures is reported in the last column.

Method	B/M	Vgg-19	ResNet-18	DenseNet-121	Avg relevancy	std relevancy	Avg Acc
ProtoPNet	B	87.50	91.67	97.92	**92.36**	5.24	**74.34**
PCPPN (k=2)	B	89.58	93.75	91.67	91.67	**2.08**	73.22
ProtoPNet	M	4.17	81.25	70.83	52.08	41.82	89.34
PCPPN (k=2)	M	83.33	91.67	87.50	**87.50**	**4.17**	**96.90**
ProtoPNet	all	45.83	86.46	84.38	72.23	22.88	83.42
PCPPN (k=2)	all	**86.46**	**92.71**	**86.58**	**89.58**	**3.12**	**87.56**

From pathologists’ point of view, the most accurate CAD system is not necessarily the most desirable one, and they prefer the one that offers a good balance of accuracy and interpretability. Considering the accuracy and relevancy criteria, and also concepts of the disagreement between pathologists, repeatability, and reproducibility, it was shown that by leveraging the PCPPN method with $K=2$ clusters we achieve the most accurate and reliable prototype-based interpretable CAD system for the digital pathology application.

### Performance of ProtoPNet in digital pathology

**Figure 3:**
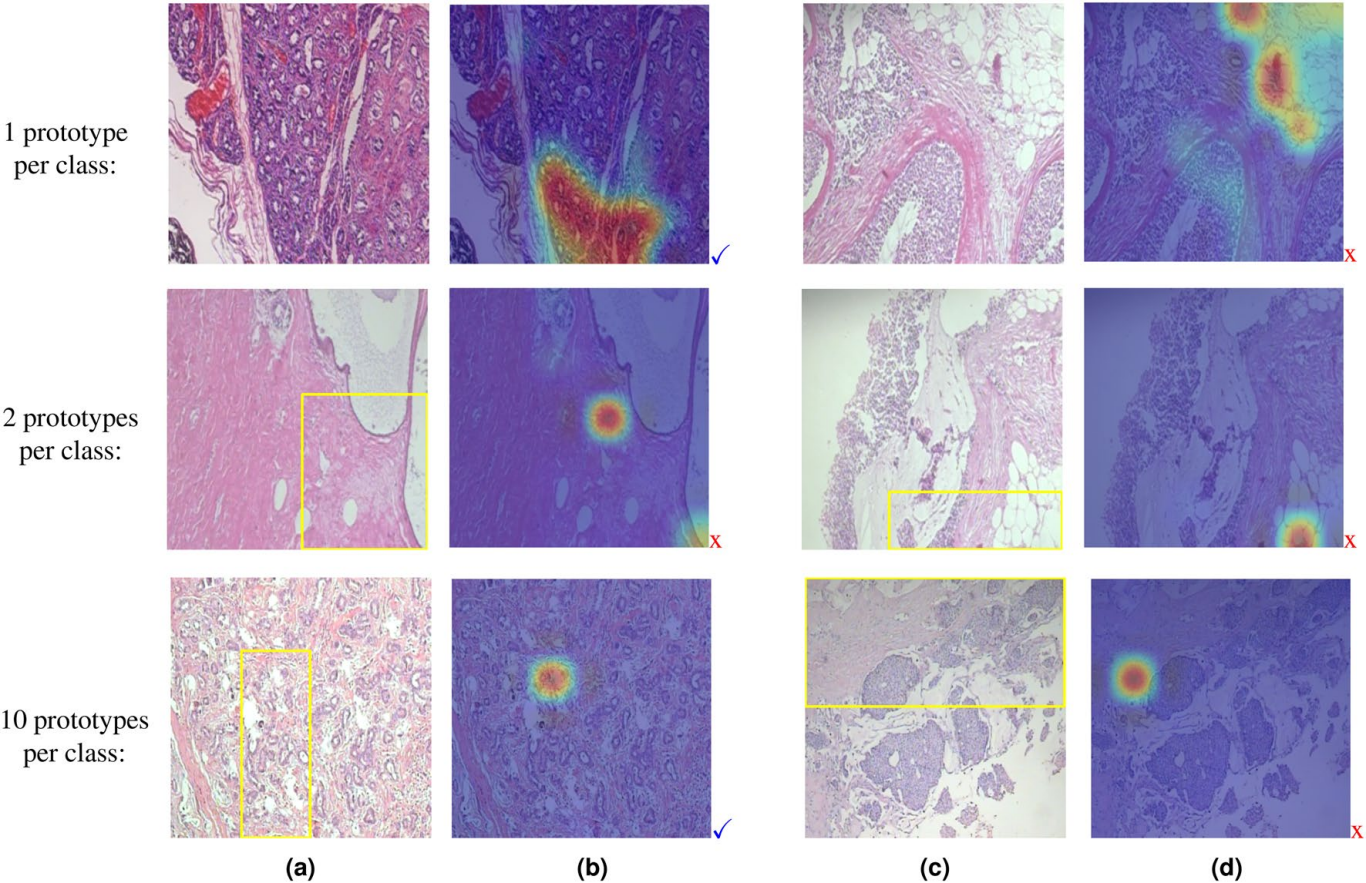
Learned prototypes by ProtoPNet for histopathology image classification when a different number of prototypes are used. (**a**) first prototype of the class benign and (**b**) its activation map, (**c**) first prototype for the class malignant, and (**d**) the corresponding activation map. The learned prototypes are highlighted by yellow boxes in the corresponding training images. Symbols $\checkmark$ and x denote respectively medically relevant and irrelevant as determined by pathologists.

To get an overview of the performance of ProtoPNet in the digital pathology application, we conducted an experiment on fold 1 in the dataset. We investigate the characteristic of the learned prototypes when different numbers of prototypes are considered in the ProtoPNet structure. Figure 3 shows the first learned prototypes for the benign and malignant breast cancers along with the corresponding activation maps—which indicates the contribution of images’ pixels in the prototype—for different number of prototypes per class (1, 2, and 10). Although the model has a fairly high test accuracy score of around 85%, with just 1 prototype per class, it can be seen that the learned prototype for the malignant class is not appropriate and it uses quite irrelevant medical information for making correct predictions. Also, when we use 2 prototypes per class, 3 out of the 4 learned prototypes are irrelevant and only one prototype of the benign class is meaningful, and yet the test accuracy is fairly high. Increasing the number of prototypes to 10 per class leads to the same results for accuracy, but from the interpretability perspective, we found that most of the learned prototypes, (8 prototypes per class), are duplicated and there are only 2 unique prototypes for each class. However, the prototypes of the malignant class are still irrelevant. Hence, it was concluded that the ProtoPNet fails to deal with histopathology images, in particular images of the malignant class which are more important. Overall the results were deemed unacceptable by pathologists since the provided explanations reveal that the model exploits mostly irrelevant information in the reasoning process. Thus, the necessity of managing the confounding issue is quite obvious for ProtoPNet to be integrated into real-world breast cancer classification CADs for digital pathology.

### Effect of the number of classes on ProtoPNet

In order to further validate the motivation behind the proposed method for improving the confounding issue of the ProtoPNet, and also to have a better insight into what causes this issue, we investigated the effect of the number of classes on the performance of ProtoPNet in a real-world application. To this end we choose the application of the bird species classification, i.e. UB-200-2011 dataset^42^, where there are a large number of classes (200 bird species), and hence it enables studying the effect of the number of classes.

**Figure 4:**
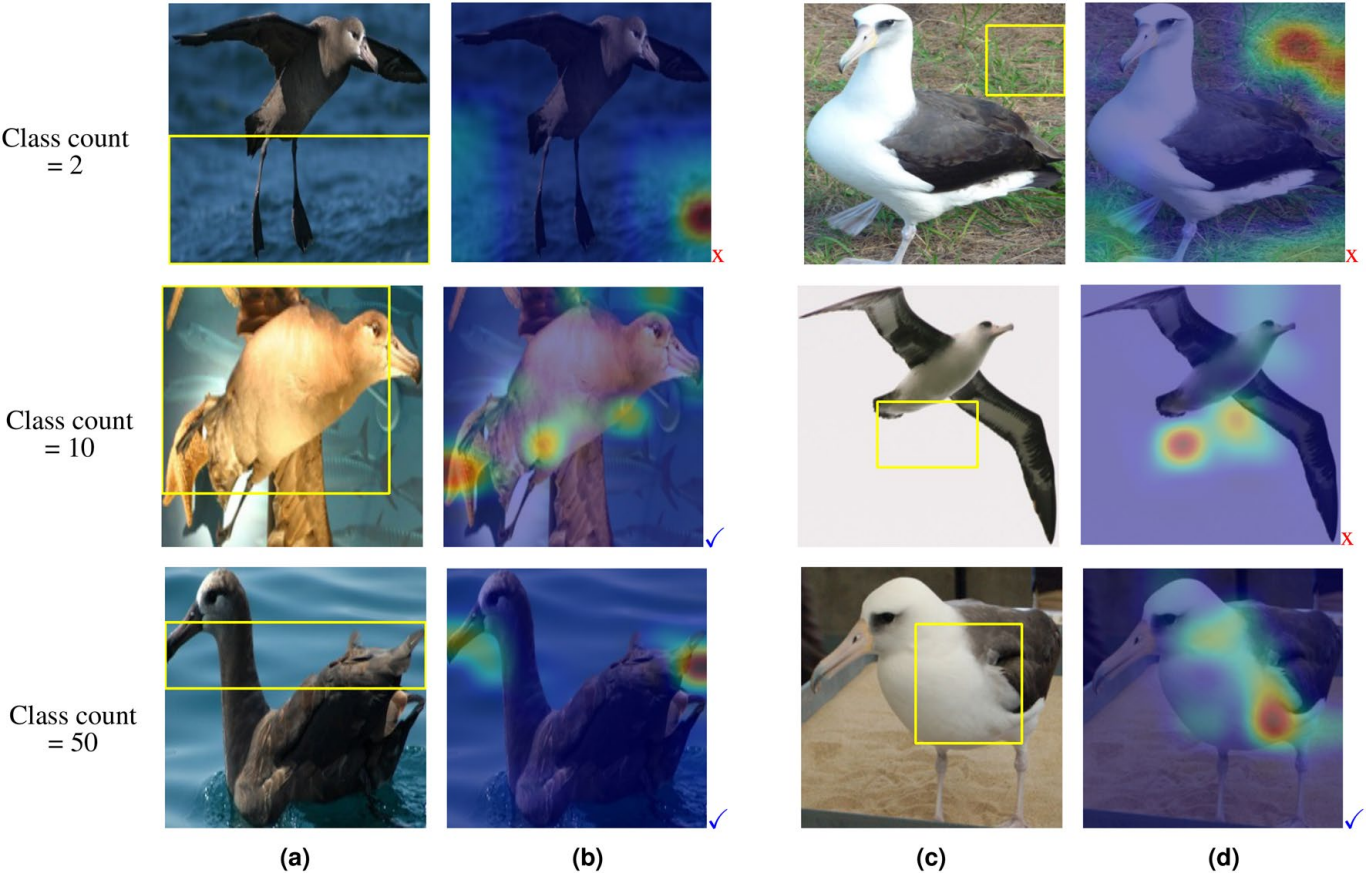
Examples of learned prototypes by ProtoPNet for bird species classification among subsets of the CUB dataset with different numbers of classes. (**a**) first learned prototype for class 1 and (**b**) corresponding activation map, (**c**) first learned prototype for class 2, and (**d**) corresponding activation map. The learned prototypes are highlighted by yellow boxes in the corresponding training images. Signs $\checkmark$ and x stand for relevant and irrelevant information, respectively.

Figure 4 shows the learned prototypes for the first 2 classes corresponding to the bird species named ’Black-Footed-Albatross’ and ‘Laysan-Albatross’, through the image classification of three different subsets of CUB-200-2011 dataset^42^, including the first 2, 10, and 50 classes of the dataset. From the first row of Fig. 4, we can see that when ProtoPNet is dealing with just 2 classes, it considers quite irrelevant parts of the images for making its predictions. When the model is trained for classifying 10 species, the learned prototype of class 1 is fairly relevant (middle row). Interestingly, from the last row of Fig. 4, we see that increasing the number of classes to 50 leads to quite relevant prototypes. Moreover, to investigate the effect of the number of classes in the duplicated prototypes, we increased the number of prototypes per class in the two experiments with 2 and 50 classes. We realized when classifying 2 bird species, there are only two unique prototypes per class (which are irrelevant of course) and the rest are duplicates. However, when classifying 50 species all learned prototypes are unique and fairly relevant.

From these observations, we can conclude some key findings including (1) The number of duplicate learned prototypes decreases as the number of classes increases, thus the model can efficiently use the potential of employing more prototypes to catch more information from the learning dataset and improve its performance. (2) Increasing the number of classes makes the learned prototypes more meaningful. Dealing with a small number of classes, ProtoPNet finds some irrelevant regions of the image like the background as discriminative features. This fact comes back to the nature of the optimization problem of the model which forces the model to correctly map each sample of the training data to the corresponding label as much as possible. Through this process what the model utilizes is only the error signal obtained from the difference between its predictions and the true labels, and there is no supervision of what features the model should learn or what parts of the image it should pay attention to. So, when the classification task has a small number of classes and the variety of images in each class is not so high, the ProtoPNet model finds the simplest discriminative features between classes that are usually some irrelevant parts of images like the background. But, on the contrary, when there are a large number of classes in the dataset or the images have a wide variety per class, the problem gets more complicated and those irrelevant parts will not be discriminative for classes anymore, hence ProtoPNet is forced to find more meaningful features. As a result, we noticed that the ProtoPNet architecture cannot extract meaningful prototypes in applications with a small number of classes, which in turn limits its use in many medical applications where the number of classes is often small. Inspired by these findings, we proposed a novel training method that implicitly increases the number of classes by producing some pseudo-classes.

### Implementation details

The model is trained in three distinct stages. First, training the convolutional layer and the prototypes in which the model aims to provide a clustering-like embedding space for facilitating the classification based on the *l*2 distance. To this end, the weights of the convolution layer $\varvec{W}$ and prototype layer are learned through the training set $D=\{(x_i,y_i) \}_{i=1}^n$ to solve the Eq. (1). In the second stage, each prototype is replaced by the nearest training patch in latent space. Eventually, the weights of the fully connected layers are adjusted so that they minimize the equation (1) accompanied by a regularization term which forces them to take near-zero values for links between each prototype and output neuron of opposite classes (see^29^ for more details). Throughout our experiments, in the clustering stage, the Resnet-18 structure, pre-trained on ImageNet, was trained on the train images of the BreakHis dataset independently and used to form a suitable latent space. The training process is done using the stochastic gradient descent (SGD) method with Adam optimizer and the initial learning rate of 0.001. By clustering the training data of each class, they are re-labeled with $2 \times K$ pseudo-classes. Dealing with a categorical classification problem with $2 \times K$ classes, the original structure of ProtoPNet^29^ with the pre-trained VGG-19 as its convolutional backbone was used. All other hyper-parameters were set to their default values following^29^. The training data of each pseudo-class were augmented such that the training samples of each main class reach 4800 images in total. Then the model was trained for 30 epochs. Finally, after the model converged, the network’s outputs were remapped to the benign and malignant classes.

## Conclusion

In this paper, we adopted the well-known interpretable prototype-based network, ProtoPNet, for breast cancer diagnosis via digital histopathology images of the BreakHis dataset. We demonstrated its failure in this application, arising from the confounding issue of the model. Dealing with the confounding issue, we proposed PCPPN. It is based on the clustering concept and does not need pixel-level annotated data. It first brings out the existing hidden structures of the training data and then re-labels the images to some new pseudo-classes within each class. In this way, the proposed method implicitly increases the number of classes and forces the model to use more relevant regions of the images in its decision-making. For evaluation, in addition to the test accuracy, we defined a new relevancy metric by exploiting the comments of a team of specialists to assess the level of interpretability. Experimental results confirmed the effectiveness of the proposed method in improving both the classification accuracy and interpretability, respectively, by $8 \%$ and $18 \%$ compared to the ProtoPNet.

## Data Availability

The breast cancer histopathological images (BreakHis dataset) and the bird species images (CUB-200-2011 dataset) used in the current study are available at https://www.kaggle.com/datasets/ambarish/breakhis and https://www.vision.caltech.edu/datasets/cub_200_2011/, respectively. The implementation code is available at https://github.com/MA-Choukali/PCPPN.
